# Tibial tubercle osteotomy decreases femorotibial rotation in patients with patellar instability

**DOI:** 10.1007/s00402-025-06023-3

**Published:** 2025-08-08

**Authors:** Martin Hartmann, Jakob Ackermann, Niklas Bergheim, Lazaros Vlachopoulos, Florian Imhoff, Lukas Jud, Sandro Fucentese

**Affiliations:** 1 Universitätsklinik Balgrist, Zurich, Switzerland; 2 University Hospital of Basel, Basel, Switzerland

**Keywords:** Tuberosity osteotomy, Trochleoplasty, Femorotibial rotation, MPFL reconstruction, Patellar instability

## Abstract

**Background:**

Patellar instability is a multifactorial pathology that poses significant challenges for orthopaedic surgeons in accurately diagnosing and effectively addressing its underlying causes. Recently, increased femorotibial (FT) rotation has been shown to contribute to patellar instability by further lateralizing the muscle force vector acting on the patella. However, there is a paucity of evidence regarding interventions that influence this parameter.

**Hypothesis/purpose:**

To assess whether patellar stabilizing procedures influence FT rotation in patients with trochlear dysplasia (TD) in the setting of patellar instability. It was hypothesized that tibial tubercle osteotomy (TTO) reduces FT rotation by changing the vector acting on the proximal tibia.

**Study design:**

Retrospective cohort study, level of evidence 3.

**Methods:**

One-hundred-forty-four knees who underwent patellar stabilizing surgery between January 2010 and December 2020 were retrospectively analysed. Caton-Deschamps index (CDI), tibial-tubercle-trochlear-groove distance (TTTG), tibial tubercle (TT) torsion, tibial tubercle-to-posterior cruciate ligament distance (TT-PCL), and pre- and postoperative FT rotation were assessed. Based on the performed patellar stabilizing procedures, knees were stratified into 4 groups: 1: Isolated medial patella-femoral ligament (MPFL) reconstruction (*n* = 51), 2: MPFL reconstruction and TTO (*n* = 24), 3: MPFL reconstruction and trochleoplasty (*n* = 37), 4: MPFL reconstruction, trochleoplasty, and TTO (*n* = 32).

**Results:**

Preoperative FT rotation differed significantly between groups (-0.2 ± 6.1° vs. 3.1 ± 6.7° vs. 5.0 ± 5.6° vs. 9.6 ± 6.0°, *p* < 0.001). Group 4 showed a significant reduction of FT rotation postoperatively, indicating a decrease in external rotation (ΔFT rotation: -2.0 ± 3.5°, *p* = 0.003). Group 1, 2 and 3 showed no reduction of FT rotation (group 1: 0.6 ± 4.8°; group 2: -1.3 ± 7°, group 3: 0.0 ± 5.3°, n.s.). Comparing knees with and without TTO, those with concomitant TTO (groups 2 and 4; *n* = 56) showed a significantly reduced postoperative FT rotation by a mean of 1.7 ± 5.3° compared to knees without TTO (0.3 ± 5°, groups 1 and 3; *n* = 88) (*p* < 0.021). The reduction in FT rotation significantly correlated with the reduction of the TT torsion but not with the medialization achieved by TTO (*r* = 0.511, *p* < 0.001 and *r* = 0.185, *p* = 0.173, respectively).

**Conclusion:**

Tibial Tubercle Osteotomy effectively reduces femorotibial rotation in patients with patellar instability and trochlear dysplasia. This reduction is directly associated with the decrease in tibial tubercle torsion. Therefore, TTO should be considered for patients with increased TT-TG distance and elevated femorotibial rotation to improve patellar stability outcomes.

## Introduction

Patellar instability remains a challenging pathology of the knee joint. Approximately 3% of all knee injuries involve patellar dislocations with an incidence ranging from 5.8 to 43 per 100,000 people [13, 22]. The conventional primary intervention for initial patellar dislocation without chondral injury is nonoperative management [7, 26]. Nonetheless, recurrence rates ranging from 15 to 44% have been reported [10]. If patellar instability persists, surgical intervention may be considered. Depending on the anatomical predispositions of each individual patient, operative care must be carefully planned and tailored to address the underlying anatomical causes [14, 18]. 

Numerous patient-specific anatomical predispositions have been identified including trochlear dysplasia (TD), patella alta, increased tibial tubercle trochlear groove (TTTG) distance, increased patella tilt (PT), disruption of the medial patellofemoral ligament (MPFL), valgus leg axis, and increased femoral anteversion [8, 13, 28]. It has also been shown that increased tibial tubercle (TT) torsion, which refers to the tilt of the tuberosity in relation to the femoral axis, is another risk factor [5]. In a recent study, TT torsion was the strongest predictor of patellofemoral instability, with patients exhibiting TT torsions of 17.7° or more having a 55-fold higher risk of developing patellofemoral instability [20]. 

In recent years, there has been an increased focus on femorotibial (FT) rotation as an elevated FT rotation (i.e., elevated external rotation of the tibia relative to the femur) has been linked to patellar maltracking in individuals experiencing patellar instability [3, 11, 20, 23, 30]. An increased FT rotation leads to an apparent lateralization of the tibial tuberosity and, consequently, to an increased TTTG distance. This results in an enhanced lateral force vector acting on the patella, which increases the risk of lateral patellar instability.

Remarkably, Wu et al. demonstrated that a TTTG distance greater than 20 mm, which is frequently used as a threshold for tibial tubercle osteotomy (TTO), was primarily attributable to increased FT rotation [30]. Given these findings, FT rotation should be taken into account—and potentially addressed—when planning surgical treatment for patellar instability. At the same time, the growing recognition of FT rotation raises important questions about its clinical management. However, concrete strategies for its correction are still lacking, as current approaches remain hypothetical and have yet to be validated by empirical studies [19]. 

Both TTO and trochleoplasty serve to realign the muscle vector acting on the patella and, consequently, the proximal tibia by reducing the TT-TG distance—although this is not the primary objective of either procedure, both contribute to its reduction [12, 15]. This leads to the theoretical proposition that TTO may attenuate FT rotation by inducing internal rotation of the tibia. This effect is potentially explained by the altered line of pull of the quadriceps muscle, which, following a medializing TTO, inserts more medially—thereby reducing the FT rotation.

Hence, the goal of the current study was to assess whether patellar stabilizing surgery influences FT rotation in patients with trochlear dysplasia undergoing surgical treatment for patellar instability. It was hypothesized that TTO significantly reduces FT rotation in patients undergoing patellar stabilizing surgery in the setting of TD.

## Methods

Ethical approval was granted by the local research ethics committee, and all included patients gave their written consent. (BASEC-Nr. 2020-01052).

### Patients

This retrospective single-center study reviewed 401 knees with TD that underwent patellar stabilizing surgery between January 2010 and December 2020. Indications for patellofemoral stabilizing surgery included symptomatic patellar instability unresponsive to nonoperative treatment for at least 3–6 months. Exclusion criteria included knees without pre- and postoperative MRI and those with prior bony procedures on the affected knee. Consequently, 144 knees remained in the study and were categorized into four groups based on the type of surgery performed.

Group 1: Isolated MPFL reconstruction (*n* = 51). Group 2: MPFL reconstruction and TTO (*n* = 24). Group 3: MPFL reconstruction and trochleoplasty (*n* = 37). Group 4: MPFL reconstruction, trochleoplasty, and TTO (*n* = 32).

Importantly, lateral lengthening was performed in all cases undergoing MPFL reconstruction. Lateral lengthening involved a Z-plasty of the lateral retinaculum by incising its superficial and deep layers at staggered positions. After controlled lengthening, both layers were sutured, preserving the integrity of the lateral stabilizers. For the MPFL reconstruction, an autologous gracilis tendon was used as the graft, anatomically fixed to the medial patellar border and the femoral origin (Schöttle point) using interference screws.

TTO involves creating an osteotomy of the tibial tubercle, which is then shifted medially. The osteotomized fragment was fixed with screws.

Trochleoplasty was performed using the thin-flap Bereiter technique, a deepening procedure designed to treat severe trochlear dysplasia. In this method, a U-shaped osteochondral flap is created in the trochlear groove, the underlying subchondral bone was carefully removed to deepen the groove, and the flap was then molded and fixed into the new, deeper position.

The indications for which surgical procedure(s) or combination of these were performed were determined individually for each patient, with no absolute criteria applied; each treatment decision was tailored to the multifactorial causes of the specific instability based on the patient’s combination of risk factors.

### Clinical and radiographic assessment

All MRI examinations were performed at our institution. Patients were positioned uniformly and examined using the same knee coil, ensuring standardized knee flexion during image acquisition. The orientation of the MRI planes was also consistently calculated using the same method.

The medical histories, clinical notes and surgical reports of patients undergoing patellar stabilization procedures were reviewed to determine age, sex, body mass index (BMI) and Dejour type of TD. Additionally, two independent readers (M.H. and N.B.), who were blinded to the surgical groups and the pre- and postoperative status, analyzed the following parameters in the MRIs: Caton-Deschamps index (CDI), TTTG distance, TT torsion, tibial tubercle-posterior cruciate ligament (TT-PCL) distance and FT rotation.

CDI is calculated by dividing the distance between the anterior angle of the tibial plateau and the most inferior aspect of the patellar articular surface by the length of the patellar articular surface [4]. TTTG distance was assessed between the most anterior point of the tibial tuberosity (TT) and the deepest point of the trochlear groove, perpendicular to the tangent to the bony borders of the posterior condyles on axial scans [16, 24]. As TT positioning involves both transverse lateralization and tilt due to external torsion around a craniocaudal axis, the TT-torsion relative to the posterior femoral condylar plane was also assessed [5, 20]. The TT-PCL distance is the mediolateral measurement between the midpoint of the TT and the medial border of the posterior cruciate ligament. This measurement was taken parallel to the dorsal tibial condylar line [25]. 

**Fig. 1:**
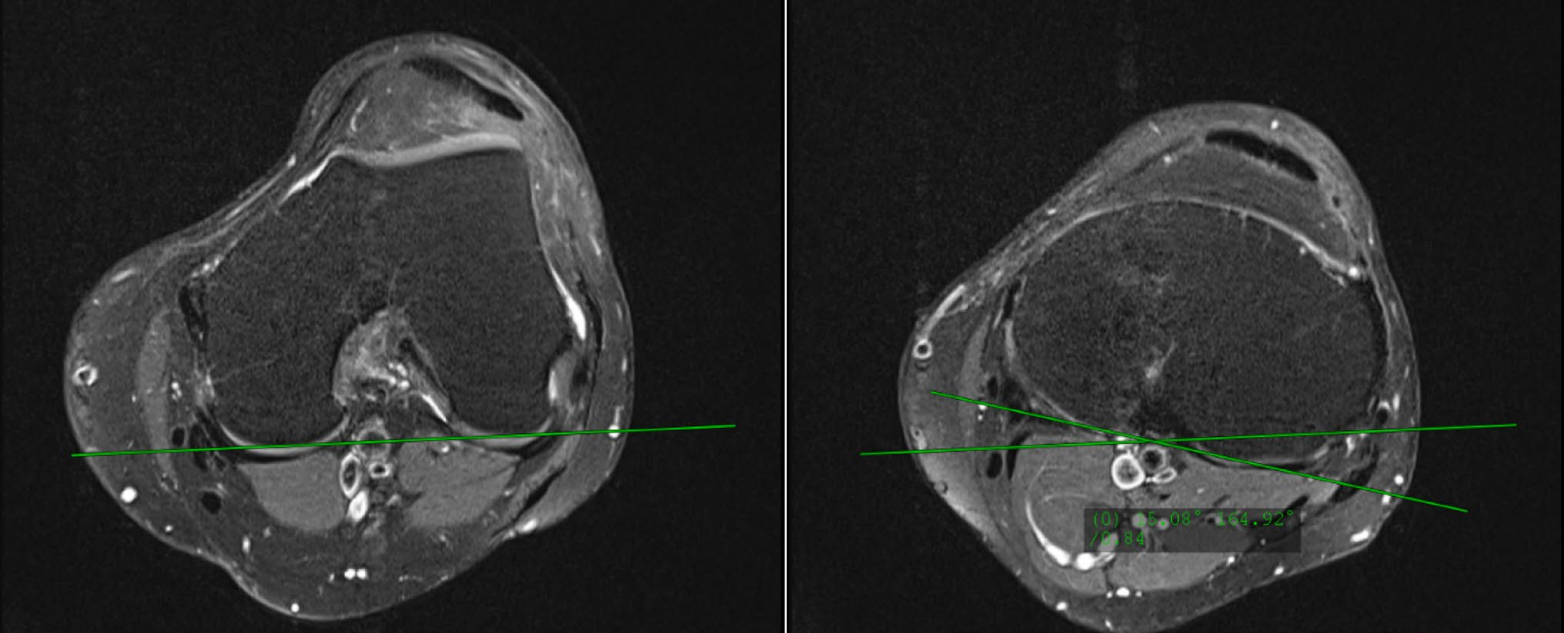
Measurement of femorotibial (FT) rotation

FT rotation was measured on axial images between the tangent of the posterior femoral condyles and the tangent of the posterior tibial plateau [29]. A positive angle indicates external rotation of the proximal tibia relative to the distal femur (Fig. 1) [9]. 

All images were read independently on a picture archiving and communication system (PACS) workstation certified for clinical use (MERLIN 7.1.22, Phönix-PACS GmbH).

### Statistical analysis

Sociodemographic and clinical characteristics of patients were determined using descriptive statistics. Continuous variables are reported as means and standard deviations (SD). Intraclass correlation coefficients with 95% confidence intervals (CI) were calculated for all measurements. Normality of distribution was tested using the Shapiro-Wilk test. Accordingly, parametric and non-parametric tests were applied to assess differences between pre- and postoperative anatomical parameters, as well as differences between groups, with Bonferroni correction applied. Pearson correlation was used to evaluate the influence of anatomical parameters on FT rotation. Multivariate linear regression was used to assess the effect of TTO on ΔFT rotation, adjusting for other procedures and preoperative FT rotation. A subgroup analysis was performed for patients with high FT rotation.

All statistical analyses were performed in SPSS for Mac (Version 23.0, SPSS Inc., Chicago, Illinois). Significance was set at *p* < 0.05.

A post-hoc power analysis showed that with a sample size of 56 patients with TTO and 88 patients without TTO, the study had a power of 0.35 to detect a difference in FT rotation on a level of significance of 0.05. Power calculation was performed with G*Power version 3.1.

## Results

On average, patients were 23.6 ± 8.4 years old and had a BMI of 25 ± 5 kg/m^2^. Twenty-five knees (17.4%) presented with TD Dejour type A, 48 (33.3%) with type B, 39 (27.1%) with type C, and 32 (22.2%) with type D.

Intraclass correlation coefficients (ICCs) for all measured parameters ranged between 0.724 and 0.914 (*p* < 0.001) (Table 1).

In an initial analysis, all four groups were compared to each other (Table 2). Among the groups, no statistically significant difference was observed regarding age (*p* = 0.631) and BMI (*p* = 0.739). In the preoperative baseline values, Group 1 exhibited a significantly higher TTTG distance compared to the other groups. A significant difference in TT-PCL distance was only observed between Group 2 and Group 3. However, preoperative FT rotation differed significantly between groups (-0.2 ± 6.1° vs. 3.1 ± 6.7° vs. 5.0 ± 5.6° vs. 9.6 ± 6.0°, *p* < 0.001). Group 4 showed a significant reduction in FT rotation postoperatively (delta FT rotation: -2.0 ± 3.5°, *p* = 0.003). Group 1, 2 and 3 showed no reduction of FT rotation (group 1: 0.6 ± 4.8°; group 2: -1.3 ± 7°, group 3: 0.0 ± 5.3°, n.s.).

In a subsequent step, as part of a subanalysis, the groups with and without concomitant TTO (group 2 + 4; *n* = 56 knees and group 1 + 3, *n* = 88 knees, respectively) were compared (Table 3). While knees without TTO showed an unchanged pre- and postoperative FT rotation (0.3 ± 5°, n.s.), postoperative FT rotation was significantly reduced by a mean 1.7 ± 5.3° in knees that underwent concomitant TTO (*p* < 0.021). Reduction of FT rotation was significantly correlated with the reduction in TT torsion but not with the medialization achieved by TTO (*r* = 0.511, *p* < 0.001 and *r* = 0.185, *p* = 0.173). Delta FT rotation was also directly associated with the degree of preoperative FT rotation (*r* = 0.316, *p* = 0.018).

In a multivariate regression model including all surgical procedures (MPFL reconstruction, TTO, and trochleoplasty), TTO was an independent predictor of change in FT rotation (β = -2.0; 95% CI: -3.7 – -0.2; *p* = 0.028). However, after adjusting for preoperative FT rotation, this association was no longer statistically significant (β = -1.0; 95% CI: -2.7–0.8; *p* = 0.271). Notably, in a subgroup analysis including only patients with elevated preoperative FT rotation (≥ 8°, *n* = 41), TTO was again significantly associated with a greater reduction in FT rotation (β = -3.0; 95% CI: -5.7 – -0.3; *p* = 0.031).

**Table 1:** Intraclass correlation coefficients for all assessed parameters

	ICC	95% CI	*p*-Value
CDI			
Preoperative	0.851	0.793–0.893	< 0.001
Postoperative	0.862	0.809–0.901	< 0.001
TT torsion, °	0.896	0.855–0.925	< 0.001
Preoperative	0.844	0.784–0.888	< 0.001
Postoperative			
TTTG distance, mm			
Preoperative	0.914	0.881–0.938	< 0.001
Postoperative	0.887	0.842–0.918	< 0.001
TT-PCL distance, mm			
Preoperative	0.821	0.752–0.872	< 0.001
Postoperative	0.724	0.616–0.801	< 0.001
Femorotibial rotation, °			
Preoperative	0.832	0.767–0.879	< 0.001
Postoperative	0.859	0.805–0.899	< 0.001

CDI = Caton-Deschamps-Index; TT = Tibial tubercle; TTTG = Tibial-Tubercle-Trochlea-Groove distance; TT-PCL = Tibial-Tubercle-Posterior-Cruciate-Ligament distance; n.s. = not significant

**Table 2:** Patients demographics and assessed radiological parameters

	Group 1 (MPFL)	Group 2 (MPFL + TTO)	Group 3 (MPFL + Trochleoplasty)	Group 4 (MPFL + TTO + Trochleoplasty)	*p*-Value
Age	22.6 ± 8.0	24.0 ± 10.5	24.5 ± 8.4	24.2 ± 7.5	n.s
BMI	24.8 ± 5.4	24.3 ± 3.9	25.1 ± 5.6	25.8 ± 4.6	n.s
CDI					
Preoperative	1.1 ± 0.1	1.1 ± 0.2	1.1 ± 0.2	1.1 ± 0.2	n.s
Postoperative	1.0 ± 0.1	1.0 ± 0.2	1.0 ± 0.2	1.0 ± 0.1	n.s
TT torsion, °					
Preoperative	12.7 ± 6.8	15.9 ± 7.3	18.4 ± 6.2	21.7 ± 5.8	< 0.001
Postoperative	12.8 ± 6.3	11.2 ± 8.3	16.1 ± 6.7	16.6 ± 5.3	0.003
TTTG distance, mm					
Preoperative	10.8 ± 3.3	15.2 ± 3.9	13.8 ± 3.3	17.2 ± 3.3	< 0.001
Postoperative	11.5 ± 3.9	7.6 ± 4.9	14.0 ± 4.1	8.7 ± 4.7	< 0.001
TT-PCL distance, mm					
Preoperative	18.5 ± 3.5	20.4 ± 3.6	17.7 ± 2.8	19.1 ± 3.1	0.015
Postoperative	17.8 ± 3.4	12.9 ± 4.8	17.8 ± 3.3	12.3 ± 3.9	< 0.001
Femorotibial rotation					
Preoperative	-0.2 ± 6.1	3.1 ± 6.7	5.0 ± 5.5	9.5 ± 6.0	< 0.001
Postoperative	0.4 ± 6.0	1.8 ± 8.1	5.0 ± 7.3	7.5 ± 5.7	< 0.001

CDI = Caton-Deschamps-Index; TT = Tibial tubercle; TTTG = Tibial-Tubercle-Trochlea-Groove distance; TT-PCL = Tibial-Tubercle-Posterior-Cruciate-Ligament distance; n.s. = not significant

**Table 3:** Comparison of the combined groups with and without TTO

	Without TTO (group 1 + 3)	TTO (group 2 + 4)	*p*-Value
CDI			n.s
Preoperative	1.1 ± 0.2	1.1 ± 0.2	n.s
Postoperative	1.0 ± 0.2	1.0 ± 0.2	
TT torsion, °			
Preoperative	15.1 ± 7.2	19.2 ± 7.0	0.001
Postoperative	14.2 ± 6.7	14.3 ± 7.2	n.s
TTTG distance, mm			
Preoperative	12.1 ± 3.6	16.4 ± 3.6	< 0.001
Postoperative	12.5 ± 4.2	8.2 ± 4.8	< 0.001
TT-PCL distance, mm			
Preoperative	18.1 ± 3.2	19.7 ± 3.3	0.008
Postoperative	17.8 ± 3.3	12.6 ± 4.3	< 0.001
Femorotibial rotation, °			
Preoperative	2.0 ± 6.4	6.8 ± 7.1	< 0.001
Postoperative	2.3 ± 6.9	5.1 ± 7.3	0.021

CDI = Caton-Deschamps-Index; TT = Tibial tubercle; TTTG = Tibial-Tubercle-Trochlea-Groove distance; TT-PCL = Tibial-Tubercle-Posterior-Cruciate-Ligament distance; n.s. = not significant

## Discussion

The most significant and truly novel contribution of this study is the identification of a previously undescribed effect of TTO: a significant reduction in FT rotation in patients with patellar instability. To the best of our knowledge, this is the first study to demonstrate a direct association between TTO and a measurable change in FT rotation. This rotational correction was specifically linked to a reduction in TT torsion, rather than to medialization alone. Moreover, the effect was more pronounced in patients with greater preoperative FT rotation, indicating that increased baseline rotational misalignment may confer a higher potential for rotational correction through TTO.

An increased FT rotation is an acknowledged risk factor for patellar instability and may therefore be a risk factor for the subsequent development of osteoarthritis in the patellofemoral joint [9, 21, 27]. FT rotation is frequently disregarded as a contributing factor to knee pathology, receiving inadequate assessment and, consequently, limited treatment [17]. However, it has been demonstrated that an elevated FT rotation is correlated with patellar maltracking in individuals experiencing recurrent patellar dislocation. Typically, patients with increased FT rotation exhibit more pronounced patellar maltracking [30]. Therefore, preoperative diagnostics should include an evaluation not only of commonly known risk factors such as TTTG, TD and alignment but also of FT rotation. While torsion is frequently assessed in patellar instability, results of a recent study did not show an association between femoral or tibial torsion and patellar tilt (PT) or trochlear engagement index (AEI) [21], considering that both parameters are known risk factors for patellar instability [14]. This may suggest that femoral or tibial torsion are not primarily linked to patellar instability, which was also shown by Balcarek et al. [2] However, both PT and AEI were found to be significantly influenced by FT rotation and TTTG distance, which are strongly associated with each other [2, 21]. This emphasizes the significance of FT rotation and TTTG in the evaluation of patellofemoral tracking.

While an increased TTTG distance can effectively be corrected by (antero-)medializing TTO, evidence on the correction of FT rotation in patients with patellar instability is scarce. In a recently published study by Jud et al., the authors were able to show that correcting femoral anteversion by derotational distal femoral osteotomy does not affect FT rotation [19]. As increased FT rotation is frequently associated with increased TTTG, it would be desirable to surgically correct both parameters simultaneously. As the position of the tibial tubercle on the tibia influences the force vector acting on the proximal tibia, this study hypothesized that medializing the tibial tubercle and thus, shifting the acting point on the proximal tibia medially, would internally rotate the tibia relative to the distal femur, leading to a decrease in FT rotation. In fact, the current study partially proved this hypothesis by demonstrating that TTO not only decreased TTTG and TT-PCL distance but also significantly reduced FT rotation. Interestingly, however, the reduction in FT rotation was primarily attributable to the change in torsion, rather than medialization of the tibial tubercle. It is important to note that the primary goal of TTO in the current cohort was to (antero-)medialize the tibial tubercle, while the change of TT torsion was unintentional, as this was not considered an important anatomical factor during the study period. Hence, it can be postulated that the small change in FT rotation observed in this study may be due to an insufficient correction of TT torsion.

Recent other studies have investigated the effect of TT torsion on patellar instability. Chassaing et al. confirmed that internal torsion of the tibial tubercle significantly improves patellar stability. The study demonstrated that after performing a triangular internal torsion TTO of 30°, there was a significant reduction in patellar displacement and tilt, and patellar dislocation did not occur under experimental conditions [6]. In a clinical study, Jud et al. found that TT torsion is the most significant predictor of patellofemoral instability among the various assessed parameters. Their study demonstrated that TT torsion had the highest diagnostic performance, with an area under the curve (AUC) of 0.95, indicating excellent predictive accuracy. A cut-off value of 17.7° for TT torsion was identified, which provided a sensitivity of 0.87 and a specificity of 0.89 for predicting patellar instability. Patients with TT torsion ≥ 17.7° had a 55-fold increased probability of experiencing patellar dislocation [20]. 

Hence, the reported results from previous studies and the findings of the current study suggest that addressing TT torsion through TTO may significantly contribute to improving patellofemoral stability not only by decreasing patellar tilt but also reducing FT rotation. Thus, TTO may not only correct an increased TTTG distance but also decrease both TT torsion and FT rotation with a single osteotomy. Future studies are warranted to further investigate this association, which should potentially be considered in the surgical algorithm to achieve optimal results after patellar stabilization.

Furthermore, the present study shows that trochleoplasty does not have a significant impact on FT rotation. While it can be postulated that medialization and alteration of TT-torsion in TTO significantly modify the quadriceps muscle pull, leading to tibial derotation in relation to the femur, the proximal muscle vector correction in trochleoplasty may be insufficient to correct FT rotation. On average, the trochlear groove is lateralized by about 6.1 mm proximally and 2.5 mm distally in patients undergoing trochleoplasty [15], which, while having a positive impact on the TTTG distance, is not sufficient to significantly alter the force vector influencing FT rotation.

The authors acknowledge the limitations of this study. One major challenge in this retrospective cohort study is the marked variation in baseline characteristics between groups, particularly regarding preoperative factors such as FT rotation. Notably, changes in FT rotation were observed only in patients who underwent concomitant TTO. Nevertheless, these baseline differences introduce significant confounding, complicating the assessment of the true effect of each surgical intervention. Although post-hoc subgroup analyses were performed, they cannot fully account for the initial group imbalances, thereby limiting the ability to draw definitive conclusions about procedure-specific outcomes. This confounding represents a fundamental limitation inherent to retrospective cohort studies and must be considered when interpreting the results. Second, this study did not collect patient-reported outcome measures to objectify clinical outcomes, yet this study solely studied the effect of patella stabilizing surgery on FT rotation. Third, the relatively small sample size when comparing four groups may have introduced a risk of type II error. Notably, the post hoc power analysis revealed a statistical power of 0.35, which considerably limits the ability to detect true differences. This implies that non-significant findings, such as the reduction in FT rotation observed in Group 2 may, in fact, represent real effects that went undetected due to insufficient power. Both Group 2 and Group 4 (with TTO) showed a reduction in FT rotation postoperatively, but statistical significance was only reached in Group 4. This suggests that larger sample sizes are needed to confirm these potential effects. Consequently, a sub-analysis comparing patients with and without TTO was conducted.

One important limitation is that postoperative MRI scans were not systematically performed for all patients following patella-stabilizing procedures. Instead, imaging was mainly reserved for those who reported ongoing symptoms. This introduces a selection bias, as postoperative data predominantly reflect patients with persistent complaints, potentially leading to an underestimation of the overall improvement experienced by the entire surgical cohort. While this bias is consistent across all groups, it nonetheless remains a critical consideration when interpreting the findings. Furthermore, only patients with TD were included in the current study, which may limit the generalizability of the results. Yet, TD is frequently seen in patients with patellar instability and thus often encountered during patient workup for patellar stabilization. We did not consider various other risk factors, such as the vastus medialis oblique (VMO), which is an active stabilizer of the patella and can contribute to instability in cases of dysplasia or insufficiency [1]as it had not been surgically addressed in the patients included in the study. Lastly, a 2° change in FT rotation is minimal and may not be clinically significant. However, since the performed TTO aimed at medialization rather than altering TT torsion, its impact on FT rotation is limited. During the study period, tibial tubercle torsion was not yet recognized as a relevant anatomical parameter and was therefore not deliberately addressed in surgical planning. This retrospective observation is of particular importance, as it implies that a targeted correction of TT torsion during TTO might have led to an even greater reduction in femoral torsion. Such considerations could play a vital role in optimizing future surgical strategies.

## Conclusion

Tibial Tubercle Osteotomy effectively reduces femorotibial rotation in patients with patellar instability and trochlear dysplasia. This reduction is directly associated with the decrease in tibial tubercle torsion. Therefore, TTO should be considered for patients with increased TT-TG distance and elevated femorotibial rotation to improve patellar stability outcomes.

## Data Availability

No datasets were generated or analysed during the current study.

## Abbreviations

AEI: Trochlear engagement index
AUC: Area under the curve
CDI: Caton-Deschamps index
FT: Femorotibial
ICC: Intraclass correlation coefficient
MPFL: Medial patellofemoral ligament
TD: Trochlear dysplasia
TT: Tibial tubercle
TTTG: Tibial-tubercle-trochlear-groove
TT-PCL: Tibial tubercle-to-posterior cruciate ligament
TTO: Tibial tuberosity osteotomy
PT: Patella tilt
